# Perceived discrimination and self-rated health in Canada: an exploratory study

**DOI:** 10.1186/s12889-016-3344-y

**Published:** 2016-08-08

**Authors:** Janice Du Mont, Tonia Forte

**Affiliations:** 1 Women’s College Research Institute, Women’s College Hospital, 76 Grenville Street, 6th Floor, Toronto, ON M5S 1B2 Canada; 2 Dalla Lana School of Public Health, University of Toronto, Toronto, ON Canada

**Keywords:** Self-rated health, Perceived discrimination, Intimate partner violence, Prevalence

## Abstract

**Background:**

Our objective was to explore whether the link between discrimination and self-rated health status differed as a function of discrimination type, including discrimination based on ethnicity/culture, race, physical appearance (other than skin colour), religion, age, and disability.

**Methods:**

A sample of 19,422 men and women aged 15 and older was included in this study. A multivariate logistic regression analysis was used to measure the association between perceived discrimination types and self-reported health status defined as excellent/good versus fair/poor.

**Results:**

The prevalence of experiencing any discrimination in the past five years was higher among those who rated their health as fair or poor (21.8 %) compared to those who rated their health as excellent or good (14.5 %, *p* < 0.0001). After controlling for all other covariates, there was a positive association between poorer self-rated health and two of the six specific discrimination variables entered into the model: perceived discrimination based on physical appearance (other than skin colour) (OR = 1.79, 95 % CI: 1.24, 2.58) and perceived discrimination based on a having a disability (OR = 1.59, 95 % CI: 1.04, 2.41).

**Conclusions:**

Our main findings indicate that perceived discrimination based on physical appearance and disability may have an adverse impact on health. The results highlight the need for a comprehensive approach to improving health outcomes that should include policies that are targeted against specific types of discrimination.

## Background

Discrimination has been defined as “the process by which a member, or members, of a socially defined group is, or are, treated differently (especially unfairly) because of his/her/their membership of that group” based on traits as diverse as race/ethnicity, gender, sexual orientation, education, social class, religion, disability, and age [Jary & Jary (1995) as cited by 1, p. 693] [1]. A large body of research demonstrates the harmful effects of discrimination on both mental and physical health. People who perceive themselves to be victims of discrimination are more likely to suffer depressive symptoms, psychological distress, anxiety, and have a decreased sense of well-being [2–4]. Perceived discrimination has also been linked to a wide variety of physical health problems such as cardiovascular disease [2, 3], as well as potential risk factors for diseases such as smoking [2, 5–7], alcohol and drug use [2, 6–8], high blood pressure/hypertension, and obesity [2, 6].

The majority of studies that have examined the relationship of perceived discrimination to health status have focused almost exclusively on perceptions of racial or ethnic discrimination [3, 4, 6] and only a few of these were conducted in Canada [9–11]. For example, in an analysis of data from the Longitudinal Survey of Immigrants to Canada, results showed that discrimination or unfair treatment based on racial or ethnic discrimination (which also included language, accent, and religion) was an independent predictor of self-reported physical and mental health problems among new immigrants and visible minorities [9].

It is largely unknown whether discrimination attributed to other characteristics also negatively impacts health. We were able to locate only a few studies that have examined the health impacts of discrimination based on characteristics beyond race/ethnicity. [12–15]. For example, a study conducted in South Korea found that for both women and men, gender, education level and age were the main types of discrimination perceived across several contexts, such as at work or educational setting, and these experiences of discrimination were significantly associated with poor self-rated health [12]. Another study conducted in Brazil examined the relative importance of a number of different types of discrimination (i.e., discrimination based on skin color/race, class and age) and their association with mental health outcomes and found that discrimination attributed to race or class were significantly associated with higher frequencies of mental disorders. Having experienced all types of discrimination was associated with the highest odds of the presence of mental disorders [15].

Canada’s demographic composition is heterogeneous and its population differs on a number of characteristics beyond race, ethnicity, and culture including language, socioeconomic status, gender, age, religious affiliation, sexual orientation, and level of abilities. As such, it is important that investigations of discrimination reflect this diversity. Examining multiple discrimination types and their association with health status could help shed light on the different mechanisms by which discrimination harms health. Clarifying the relative importance of each type of discrimination also could aid in informing policies against specific types of discrimination.

When examining the relationship between discrimination and health, it is critical to control for a number of factors shown to be associated with poor self-rated health status. These include unhealthy behaviours, such as alcohol and drug use, socio-economic determinants of health such as low income and education, and intimate partner violence (IPV). IPV is a prevalent problem that has also been associated with discrimination [16], as well as poor mental (e.g., depression, Post-Traumatic Stress Disorder, anxiety, self-harm, and sleep disorders) and physical health (e.g., somatic disorders, chronic disorders and chronic pain, gynaecological problems, and an increased risk of contracting sexually transmitted infections) [17]. Researchers have described experiences of discrimination as creating a hostile or stressful environment which in turn may be a precursor to interpersonal violence [18]. These circumstances may then function to negatively impact health directly or indirectly by creating barriers to other social determinants of health, such as employment and education.

The objective of this study was to explore whether the link between discrimination and poor self-rated health differed as a function of discrimination type using a population-based nationally representative sample of women and men. The research question of interest was: taking into account socioeconomic and other factors known to impact health, such as engaging in unhealthy behaviours and experiences of IPV, what specific types of discrimination are associated with poorer self-rated health? Given the diversity of Canada’s population, examining the impact of multiple types of discrimination is important in understanding how to improve health outcomes.

## Methods

Data used in the analyses were drawn from Statistics Canada’s General Social Survey (GSS). The GSS is a cross-sectional, national, voluntary survey that collects information on social trends in Canada. The target population includes non-institutionalized persons aged 15 years or older, living in the ten provinces. The sample is selected using random digit dialing methods and the interviews are conducted by telephone. The questionnaire was designed using focus groups and was pilot tested to ensure respondents’ understanding of the questionnaire and the appropriateness and completeness of the response categories.

Several quality assurance measures were implemented in the data collection and processing phases. For instance, training was provided to all interviewers on survey concepts and procedures. Additionally, a number of data checks were run to flag potential errors such as inconsistent or contradictory responses or responses outside the minimum or maximum range.

In order to take into account Canadians’ experiences of IPV in the association between discrimination and health, the current study used data from the 2009 cycle, the latest year for which information on Canadians’ experiences of victimization were available. More detailed methods of the survey have been described elsewhere [16]. Ethics approval for this study was provided by the research ethics board at Women’s College Hospital (REB# 2011-0047-E).

### Independent variables

#### Perceived discrimination

Survey respondents were asked a series of questions designed to assess their perceived experiences of discrimination in the past five years. Each item started with the question: “In the past five years, have you experienced discrimination or been treated unfairly by others in Canada because of…” where discrimination means “treating people differently, negatively or adversely because of their race, age, religion, sex, etc.”. Respondents were asked to indicate Yes or No to their experiences of discrimination based on each of the following: ethnicity or culture, race or colour, religion, language, sex, physical appearance (other than skin colour), sexual orientation, age, disability, or some other reason. When asked about discrimination based on physical appearance (other than skin colour), respondents were prompted by the interviewer that physical appearance could include weight, height, hair style/colour, clothing, jewelry, tattoos and other physical characteristics excluding skin colour.

Respondents were able to indicate more than one reason for their experience of discrimination. Among those respondents with sufficient data for analysis, the number of types of discrimination experienced was calculated and used to determine whether respondents experienced discrimination based on a single reason (for example, based only on their disability status) versus discrimination based on multiple reasons. Finally, survey respondents reporting discrimination were asked if they experienced discrimination when dealing with public hospitals or health care workers.

#### Sociodemographics

Sociodemographic variables were selected for inclusion based on their established relationship with self-rated health status [19]. Characteristics included sex, age in years (15–34, 35–54, 55 and older), marital status (married/common-law, widowed/separated/divorced/single), immigration status (recent immigrant [permanent resident in Canada for less than 10 years], non-recent immigrant [permanent resident in Canada for 10 years or more], Canadian-born), visible minority status (yes, no), Aboriginal status (yes, no), highest level of education achieved (high school graduate or less, more than high school), annual household income in Canadian dollars (0–$19,999; $20,000–$49,999; $50,000 or more), frequency of religious attendance (once per week, less than once per week, not at all), region of residence (rural, urban), frequency with which a physical condition limited activities of daily living (always/often, sometimes, never), and frequency with which a psychological, emotional, or mental health condition limited activities of daily living (always/often, sometimes, never).

#### Health-related behaviours and stress

In the GSS, alcohol consumption and illicit drug use were assessed by asking respondents the frequency with which they engaged in each of the behaviours in the past month (“In the past month, how often did you drink alcoholic beverages?”; In the past month, how often did you use drugs?”). The term “drugs” did not include prescription drugs or over-the-counter medications, but referred to the use of illicit drugs and the abuse of solvents and other hazardous substances. For the present analyses, responses for alcohol and drug use were grouped as “regular use” (which included the following response options: every day, 4–6 times a week, 2–3 times a week, once a week, once or twice in the past month) and rarely or never used (which included those who indicated not in the past month and never). Stress was measured by the question, “Thinking of the amount of stress in your life, would you say that most days are…?” Response options included not at all stressful, not very stressful, a bit stressful, quite a bit stressful, and extremely stressful. For the present analyses, those who responded that their life was quite a bit stressful or extremely stressful were grouped together (categorized as “high stress”) and compared to those who indicated that their life on most days was not at all stressful, not very stressful, or a bit stressful (categorized as “low stress”).

#### Intimate partner violence (IPV)

Survey respondents were asked about their experiences of IPV by a current or former partner with whom they had had contact with in the preceding 5 years from the date of the survey. IPV was examined among respondents who were legally married or living in a common-law relationship during the time of the survey, or had contact with their former partner in the previous five years. The present analyses examined ‘any IPV’, which included having experienced physical, sexual, emotional, and/or financial IPV. More details on the definition of IPV have been described previously [16]. Survey responses were grouped as having experienced IPV in the past five years or not having experienced IPV either because they had not had contact with a current or former partner in the past five years or they had had contact, but reported experiencing no violent episodes.

### Dependent variable

The dependent variable for this analysis was self-rated health status. In the GSS, self-rated health was assessed by asking respondents: “In general, would you say your health is”…excellent, very good, good, fair, or poor. For the present analyses, self-rated health status was examined as a binary variable with good health defined as excellent, very good, or good (excellent/good) and poorer health defined as fair or poor.

### Analyses

The bivariate associations between self-rated health status and discrimination, sociodemographic, health-related behaviors and stress and IPV were assessed using Chi-squared analyses. All study variables were assessed as categorical variables. In survey sampling, non-response may result in some groups being over-represented and others under-represented. Therefore, to ensure representativeness of the Canadian population, all analyses were weighted. This technique assigns an adjustment weight to each survey respondent so that persons who are under-represented get a weight larger than 1, and those in over-represented groups get a weight smaller than 1. All data were weighted according to guidelines put forth by Statistics Canada [20] and all analyses, including totals and percentages, presented in this study are weighted values.

Logistic regression analysis was used to test the association of types of perceived discrimination on self-rated health status. All variables significantly associated with health status in the bivariate analyses at a p value of 0.10 or less, were entered into the multivariate model. For the discrimination variables, this included discrimination based on ethnicity or culture, race or colour, physical appearance (other than skin colour), religion, age, and disability. To maintain model parsimony, only significant variables were retained in the final model. We used the c-statistic—equivalent to the area under a receiver-operator curve—to examine the discriminative-ability of the final logistic regression model [21].

For the multivariate analyses, a p value of 0.05 or less was considered statistically significant and odds ratios (OR) and 95 % confidence intervals (CI) are reported. For household income, the proportion of missing data was approximately 20 %. Therefore, an unknown/not stated category was included in the analysis of this variable in order to retain the sample size. The model was checked for multicollinearity and collinearity was not found to be of concern among the variables in the model.

## Results

A total of 19,422 respondents participated in the 2009 GSS. As Table 1 indicates, the sample was equally distributed with respect to sex and age group. Most respondents were married or living in a common-law relationship at the time of the survey (62.7 % compared to 37.3 % who indicated they were widowed, separated, divorced, or single). A larger percentage of the respondents had more than a high school education (67.4 % compared to 32.6 % who had less than a high school education) and most lived in a household whose annual household income exceeded $50,000 (55.6 % compared to 5.2 % whose annual household income was 0–$19,999 and 18.6 % whose annual household income was $20,000–$49,999). A relatively small percentage of respondents identified themselves as a visible minority (13.4 %), Aboriginal (2.9 %), or a recent immigrant to Canada (5.5 %). Approximately, 1 in 5 respondents were living in a rural setting (18.7 %).

**Table 1:** Sociodemographic Characteristics of Women and Men in the 2009 General Social Survey, Weighted Number and (%)

Characteristic	Number	(%)
Sex		
Female	9,838	(50.7)
Male	9,575	(49.3)
Age group (in years)		
15–34	6,393	(32.9)
35–54	7,091	(36.5)
55 and older	5,937	(30.6)
Marital status		
Married or common-law	12,174	(62.7)
Widowed, separated, divorced, or single	7,230	(37.3)
Education		
High school or less	6,287	(32.6)
More than high school	12,984	(67.4)
Annual household income		
0 to $19,999	1,010	(5.2)
$20,000 to $49,999	3,622	(18.6)
$50,000 or more	10,806	(55.6)
Unknown or not stated	3,985	(20.5)
Visible minority		
Yes	2,581	(13.4)
No	16,642	(86.6)
Aboriginal		
Yes	571	(2.9)
No	18,850	(97.1)
Immigration status		
Recent immigrant	1,052	(5.5)
Non-recent immigrant	2,954	(15.4)
Canadian-born	15,230	(79.2)
Region of residence		
Rural	3,639	(18.7)
Urban	15,782	(81.3)

The data showed that the prevalence of having experienced any type of discrimination in the past five years in Canada was 15.3 %. The most common type of discrimination perceived was discrimination based on ethnicity or culture (5.4 %), followed by race or colour (5.1 %). Overall, 7.8 % of Canadians reported having experienced discrimination based on more than one reason.

As seen in Table 2, the prevalence of having experienced any discrimination in the past five years was significantly higher among those who rated their health as fair or poor compared to those who rated their health as excellent or good (21.8 % vs. 14.5 %, *p* < 0.0001). When specific types of discrimination were examined, the prevalence of discrimination based on a number of characteristics was significantly and consistently higher among those who rated their health as fair or poor versus excellent or good. This included discrimination based on ethnicity/culture (7.3 % vs. 5.2 %, *p* = 0.0012), race/colour (6.6 % vs. 4.9 %, *p* = 0.006), physical appearance (other than skin colour) (8.2 % vs. 3.4 %, *p* < 0.0001), religion (3.7 % vs. 2.3 %. *p* < 0.002), age (5.0 % vs. 3.1 %, *p* < 0.0001), and disability (5.1 % vs. 0.6 %, *p* < 0.0001). Among those reporting discrimination, those reporting multiple types of discrimination were more likely to rate their health poorer than excellent or good (12.8 % vs. 7.1 %). Likewise, the percentage who reported having experienced discrimination when dealing with public hospitals or health care workers was higher among those reporting fair or poor health compared to those reporting their health to be excellent or good (17.1 % vs. 10.2 %, *p* = 0.0004).

**Table 2:** Perceived Discrimination in the Past Five Years of Women and Men in the 2009 General Social Survey by Self-Rated Health Status, Weighted Number and (%)

	Self-rated health status				
	Excellent/good		Fair/poor		*p* value
	Number	(%)	Number	(%)	
Discrimination or unfair treatment based on:					
Sex	774	(4.6)	117	(5.2)	0.22
Ethnicity or culture	877	(5.2)	164	(7.3)	0.0012
Race or colour	833	(4.9)	149	(6.6)	0.006
Physical appearance (other than skin colour)	582	(3.4)	183	(8.2)	<0.0001
Religion	393	(2.3)	83	(3.7)	0.002
Sexual orientation	150	(0.9)	27	(1.2)	0.20
Age	530	(3.1)	113	(5.0)	<0.0001
Disability	105	(0.6)	114	(5.1)	<0.0001
Language	496	(2.9)	76	(3.4)	0.32
Any discrimination or unfair treatment	2457	(14.5)	485	(21.8)	<0.0001
Number of reasons for discrimination or unfair treatment^a^					
One	1235	(7.3)	198	(8.9)	<0.0001
Multiple	1204	(7.1)	284	(12.8)	
Discrimination or unfair treatment experienced when dealing with public hospitals or health care workers?^a^	234	(10.2)	75	(17.1)	0.0004

^a^Among those who reported having experienced discrimination in the past five years

Table 3 shows the bivariate relationships between self-rated health and the covariates. Women were more likely than men to have stated their health was poor or fair versus excellent or good (*p* < 0.0001), as were those who were older (*p* < 0.0001), widowed, separated, divorced, or single (*p* < 0.0001), Aboriginal (*p* = 0.003), and non-recent immigrants (*p* = 0.0001). Those with less education (*p* < 0.0001), with lower incomes (*p* < 0.0001), with more frequent activity limitations due to a physical condition (*p* < 0.0001) or a mental health condition (*p* < 0.0001), and living in rural areas (*p* < 0.0001) were also more likely to rate their health as poorer versus excellent/good. An increased likelihood of rating one’s health as poorer was also associated with reporting IPV in the past 5 years (*p* < 0.0001), reporting higher levels of stress (*p* < 0.0001), regular use of illicit drugs (*p* = 0.05), and less frequent consumption of alcohol (*p* < 0.0001).

**Table 3:** Sociodemographic and Health-Related Characteristics of Women and Men in the 2009 General Social Survey by Self-Rated Health Status, Weighted Number and (%)

Characteristic	Self-rated health status				
	Excellent/good		Fair/poor		*p* value
	Number	(%)	Number	(%)	
Sex					
Female	8,524	(87.1)	1,269	(13.0)	<0.0001
Male	8,550	(89.6)	989	(10.4)	
Age group (in years)					
15 to 34	5,951	(93.5)	413	(6.5)	<0.0001
35 to 54	6,384	(90.4)	678	(9.6)	
55 and older	4,739	(80.3)	1,166	(19.8)	
Marital status					
Married or common-law	11,000	(89.2)	1,308	(10.8)	<0.0001
Widowed, separated, divorced, or single	6,256	(86.8)	948	(13.4)	
Education					
High school or less	5,225	(83.4)	1,040	(16.6)	<0.0001
More than high school	12,000	(90.8)	1,198	(9.2)	
Annual household income					
0 to $19,999	727	(72.1)	282	(27.9)	<0.0001
$20,000 to $49,999	3,028	(83.7)	588	(16.3)	
$50,000 or more	9,952	(92.2)	847	(7.8)	
Unknown or not stated	3,368	(86.2)	541	(13.8)	
Visible minority					
Yes	2,303	(89.6)	267	(10.4)	0.14
No	15,000	(88.2)	1,965	(11.8)	
Aboriginal					
Yes	480	(84.0)	91	(16.0)	0.003
No	17,000	(88.5)	2,166	(11.6)	
Immigration Status					
Recent immigrant	977	(93.3)	70	(6.7)	0.0001
Non-recent immigrant	2,556	(86.7)	392	(13.3)	
Canadian-born	13,000	(88.3)	1,777	(11.7)	
Religious attendance					
Once per week	3,057	(87.5)	438	(12.5)	0.14
Less than once per week	6,842	(89.0)	847	(11.0)	
Not at all	7,014	(88.2)	936	(11.8)	
Region of residence					
Rural	3,112	(86.1)	503	(13.9)	<0.0001
Urban	14,000	(88.8)	1,755	(11.2)	
Daily activities limited by physical condition					
Always or often	493	(43.1)	651	(56.9)	<0.0001
Sometimes	782	(66.6)	393	(33.4)	
Never	16,000	(93.0)	1,193	(7.0)	
Daily activities limited by a psychological, emotional, or mental health condition					
Always or often	105	(47.8)	115	(52.3)	<0.0001
Sometimes	272	(64.4)	151	(35.6)	
Never	17,000	(89.5)	1,959	(10.5)	
Alcohol consumption					
Regular use	7,766	(91.8)	698	(8.2)	<0.0001
Rarely or never used	9239	(85.7)	1,541	(14.3)	
Illicit drug use					
Regular use	590	(85.1)	104	(14.9)	0.05
Rarely or never used	16,000	(88.5)	2,136	(11.5)	
Stress level, most days					
High stress	3,774	(83.3)	756	(16.7)	<0.0001
Low stress	13,000	(89.9)	1,478	(10.1)	
Intimate partner violence, in past 5 years					
Yes	2159	(84.4)	399	(15.6)	<0.0001
No	15,000	(89.7)	1825	(10.3)	

After controlling for all other covariates, findings from the multivariate logistic regression analysis showed a significant association between poorer self-rated health and two of the six specific discrimination variables entered into the model (Table 4): perceived discrimination based on physical appearance (other than skin colour) (OR = 1.79, 95 % CI: 1.24, 2.58) and perceived discrimination based on a having a disability (OR = 1.59, 95 % CI: 1.04, 2.41).

**Table 4:** Weighted Logistic Regression Analysis of Factors Associated with Self-Rated Health Status of Women and Men in the 2009 General Social Survey

Factor	Unadjusted OR (CI)	Adjusted OR (CI)
Discrimination or unfair treatment based on physical appearance (other than skin colour)	2.51 (1.97, 3.18)	1.79 (1.24, 2.58)
Discrimination or unfair treatment based on a disability	8.61 (6.35, 11.65)	1.59 (1.04, 2.41)
Age group (in years)		
15–34		Reference
35–54	1.54 (1.29, 1.82)	1.39 (1.13, 1.70)
55 and older	3.54 (3.01, 4.17)	2.64 (2.18, 3.19)
Education		
More than high school		Reference
High school or less	1.96 (1.76, 2.18)	1.59 (1.40, 1.81)
Annual household income		
0 to $19,999	4.55 (3.87, 5.35)	1.99 (1.62, 2.44)
$20,000to $49,999	2.28 (2.0, 2.61)	1.45 (1.24, 1.69)
$50,000 or more		Reference
Unknown or not stated	1.89 (1.63, 2.19)	1.39 (1.17, 1.66)
Aboriginal		
Yes	1.46 (1.14, 1.87)	1.36 (1.02, 1.80)
No		Reference
Daily activities limited by physical condition		
Sometimes	6.63 (5.66, 7.77)	4.62 (3.87, 5.52)
Always or often	17.44 (14.97, 20.32)	10.52 (8.83, 12.53)
Never		Reference
Daily activities limited by a psychological, emotional, or mental condition		
Sometimes	4.69 (3.68, 5.99)	1.95 (1.42, 2.67)
Always or often	9.29 (6.81, 12.68)	3.35 (2.21, 5.06)
Never		Reference
Alcohol consumption		
Regular use	0.54 (0.48, 0.61)	0.70 (0.61, 0.80)
Rarely or never used		Reference
Illicit drug use		
Regular use	1.35 (1.00, 1.81)	1.57 (1.08, 2.26)
Rarely or never used		Reference
Stress level, most days		
High stress	1.79 (1.59, 2.01)	1.85 (1.60, 2.15)
Low stress		Reference
Intimate partner violence, in past 5 years		
Yes	1.49 (1.30, 1.71)	1.17 (0.99, 1.38)
No		Reference

Note: c-statistic = 0.8159; *OR*, odds ratio; *CI*, confidence interval

Additional covariates significantly associated with poorer self-rated health included activity limitations due to a physical condition or a psychological, emotional, or mental health condition. Compared to those reporting no limitations in activities of daily living, those reporting limitations always or often were more likely to report poorer self-rated health (physical activity limitations OR = 10.52, 95 % CI: 8.83–12.53; psychological, emotional, or mental activity limitations OR = 3.35, 95 % CI: 2.21–5.06) as were those reporting activity limitations sometimes (physical activity limitations OR = 4.62, 95 % CI: 3.87–5.52; psychological, emotional, or mental activity limitations OR = 1.95, 95 % CI: 1.42–2.67).

Poorer self-rated health was also significantly and positively associated with older age, less education, lower income, Aboriginal status, high stress, and regular use of illicit drugs. Poorer health was negatively related to regular use of alcohol. Having experienced IPV in the past five years was marginally associated with poorer self-rated health.

## Discussion

This study examined the relationship between perceived discrimination and self-rated health status in a nationally representative sample of women and men in Canada. It provided an important opportunity to explore whether the link between perceived discrimination and poorer self-rated health differed as a function of discrimination type. We found that, even after adjusting for a number of factors known to impact health status, our results continued to show a significant and independent association between discrimination and health status. Specifically, the results identified two types of perceived discrimination to be associated with poorer self-rated health: physical appearance (other than skin colour) and disability.

Results from the multivariate analysis showed that women and men reporting having experienced discrimination based on a physical characteristic other than skin colour had a poorer assessment of their health. As physical appearance could have included a number of characteristics mentioned when respondents were asked this question (i.e., weight, height, hair style/colour, clothing, jewelry, tattoos and other physical characteristics excluding skin colour), it is unknown which particular characteristics respondents had in mind. However, research shows that discrimination based on body size weight is one of the most common forms of discrimination and has been noted in a number of different contexts including places of employment [22, 23], educational institutions [24], and health-care facilities [22, 25]. Previous work has documented an association between weight discrimination and poorer physical and mental health outcomes [26–28]. Earlier research also has shown that perceived discrimination based on weight and other issues, both outside and within health care systems, has been associated with underutilization of needed medical and mental health care [29, 30]. Headwear, jewelry, or clothing may all be visual cues to religious affiliation [31] and culture/ethnicity which also may have been the basis for the discrimination perceived.

This study found that perceived discrimination based on disability was also associated with poorer self-rated health. Previous studies have shown that people with disabilities have cited negative attitudes of health care providers as a barrier to accessing health care services [32, 33]. This is congruent with our bivariate findings that showed that approximately 1 in 5 respondents who reported poorer health also reported having experienced discrimination when dealing with public hospitals or health care workers. Studies also have found that health care providers often report feeling they do not have the appropriate training for working with people living with disabilities, including lack of disability-specific knowledge [34, 35]. Consequently, those with disabilities may not receive the appropriate treatment or preventive care they need leading to worse overall health.

Experts in the field have hypothesized that the mechanism by which discrimination may impact health is through a physiological stress response that may accumulate over time and increase one’s susceptibility to illness [3]. Individuals may cope with the stress of discrimination by adopting unhealthy behaviours such as smoking [7] and alcohol and substance use [5, 7, 8]. In the present study, our results showed that high stress in everyday life, alcohol and drug use were all independently associated with self-rated health status. However, even after adjustment for these factors, our results continued to show a significant association between specific types of perceived discrimination and poorer self-rated health. These findings suggest that factors other than those controlled for here, may explain the link between discrimination and health and highlight the need for continued research to elucidate the pathways by which discrimination negatively impacts health outcomes.

Our results showed that those reporting limitations in their daily activities due to either a physical condition or a psychological, emotional, or mental condition were more likely to rate their health as poorer. We also found a dose–response relationship between limitations in daily activities due to a physical or mental health condition and self-rated health status; those with more frequent limitations (i.e., always or often) in their activities were more likely to rate their health as poorer than excellent or good than those reporting limitations less frequently (i.e., sometimes). Previous studies have similarly documented disparities in health among those with disabilities compared to those without disabilities [36].

Our study confirms the link between a number of socioeconomic variables and self-rated health. Those with less education, lower incomes, and of Aboriginal status were more likely to rate their health as poorer. Numerous epidemiological studies demonstrate a gradient between self-rated health and socioeconomic status [37, 38]. Previous research shows that socioeconomic conditions during childhood and/or adolescence can influence the development of inequalities in health outcomes [39]. Research has also shown that compared to non-Aboriginal people, Aboriginal people are more likely to report higher rates of chronic conditions, smoke, be exposed to second-hand smoke in the home, and have higher rates of obesity, which might account for their poorer assessment of their health [40].

Our bivariate finding for IPV and its near significance in the multivariate model supports previous work that showed that having experienced IPV is associated with poor self-rated health [17]. Given the complex association among discrimination, violence and health, our results suggest that future studies on the topic of discrimination and health should control for violence in order to help untangle the relationship [16, 17]. To our knowledge, previous studies examining discrimination and health have not taken into account the role of interpersonal violence.

Our findings also showed that discrimination based on culture/ethnicity or race/colour were among the most common types of discrimination reported in our sample, and both were significantly associated with poor self-rated health in the bivariate analyses. However, despite this, they did not maintain their significance in the multivariate model. Canada has a very diverse population and much attention has been given to combating racial and cultural discrimination. Thus, although these types of discrimination continue to persist in Canada, our results suggest that we may have made some progress in circumventing their deleterious effects on health.

This study has some limitations that should be considered when interpreting the findings. First, data for this analysis are based on self-report. As such, discrimination may have been under- or over-reported across different sociodemographic groups based on differing interpretations of discriminatory experiences. The data are based on those experiences that respondents perceived as being discriminatory and may not have included those where discrimination may have been subtle or not perceived at all. Second, previous researchers have highlighted the shortcomings of using a measure of self-assessed general health in that it provides little information on what components of health the respondent is evaluating when asked to rate their health, be it, for example, vitality, social functioning, or physical functioning [19]. However, despite these noted limitations, self-rated health has been shown to be a strong predictor of morbidity [41] and mortality [42, 43]. Third, the multivariate result on the link between alcohol consumption and health needs to be interpreted with caution. While results showed that alcohol consumption was protective against poorer health, our analysis grouped moderate drinkers together with those who drank more heavily. Grouping this variable differently did not alter the finding; however, this result should be investigated further. Finally, given the cross-sectional nature of the GSS, self-rated health was measured concurrently with perceptions of discrimination and we are therefore unable to determine whether the discrimination preceded or followed respondents’ assessment of their health as poorer. It should be noted though that respondents were asked about their experiences of discrimination in the past five years, whereas respondents were asked to rate their current health status; therefore, this limitation may have been partially addressed by the design of the survey.

## Conclusion

While there has been much advancement in understanding discrimination and its role in affecting health, previous work in the area has focused primarily on racial/ethnic discrimination. To our knowledge, this study was the first in Canada to explore the relative importance of different discrimination types on self-rated health using a nationally representative dataset. The results highlight that while ethnic, cultural, and racial discrimination continue to be the most prevalent types of discrimination reported, there is a need to address other types of discrimination as part of a larger strategy to improve health outcomes. Future research should corroborate these findings but also use them to gain a better understanding of the mediators and pathways by which discrimination negatively impacts health. This research would improve our understanding of the role of discrimination as a determinant of health. In the meantime, our findings help clarify the relative importance of different types of discrimination on health outcomes which could aid in the development of targeted policies and practices.

## Abbreviations

CI, confidence interval; GSS, general social survey; IPV, intimate partner violence; OR, odds ratio
